# Elevated DLL3 in stomach cancer by tumor-associated macrophages enhances cancer-cell proliferation and cytokine secretion of macrophages

**DOI:** 10.1093/gastro/goab052

**Published:** 2021-11-25

**Authors:** Jian-Bin Ye, Jun-Jie Wen, Dan-Lin Wu, Bing-Xin Hu, Mei-Qun Luo, Yan-Qing Lin, Yun-Shan Ning, Yan Li

**Affiliations:** 1 School of Laboratory Medicine and Biotechnology, Southern Medical University, Guangzhou, Guangdong, P.R. China; 2 Service Union Medicine, Southern Medical University, Zhuhai, Guangdong, P.R. China

**Keywords:** stomach cancer, DLL3, macrophages, Notch signaling, proliferation, cytokines

## Abstract

**Background:**

The notch signal pathway is important in the development of both tumor-associated macrophages (TAMs) and stomach cancer, but how Notch signaling affects TAMs in stomach cancer is barely understood.

**Methods:**

The expressions of Notch1, Notch2, Notch3, Notch4, hes family bHLH transcription factor 1 (Hes1), and delta-like canonical Notch ligand 3 (DLL3) were detected by Western blot and the expressions of interleukin (IL)-10, IL-12, and IL1-β were detected using enzyme-linked immunosorbent assay after the co-culture of macrophages and stomach-cancer cells. The proliferation and migration of cancer cells were detected using 3-(4,5-dimethylthiazol-2-yl)-2,5-diphenyltetrazolium bromide assay and scratch assay, respectively, and the cell cycle was detected using Annexin V/propidium iodide assay. The protein interactions with DLL3 were detected using co-immunoprecipitation and mass spectrometry.

**Results:**

The co-culture of macrophages and stomach-cancer cells MKN45 and BGC823 could enhance cell proliferation accompanied by the activation of Notch1/Notch2 signaling and upregulation of DLL3. Notch signaling gamma-secretase inhibitor (DAPT) blocked this process. The overexpression of DLL3 in stomach-cancer cells could promote the proliferation of cancer cells, enhance the activation of Notch1/Notch2 signaling, induce the expression of IL-33, lead to the degradation of galectin-3–binding protein (LG3BP) and heat shock cognate 71 kDa protein (HSPA8), and result in elevated IL-1β, IL-12, and IL-10 secretion by macrophages. Higher expression of DLL3 or IL-33 could lead to a lower survival rate based on University of California, Santa Cruz Xena Functional Genomics Explorer and The Cancer Genome Atlas data set.

**Conclusions:**

This is evidence that DLL3 regulates macrophages in stomach cancer, suggesting that DLL3 may be a novel and potential target for stomach-cancer therapy.

## Introduction

Stomach cancer ranked as fifth for cancer incidence and third for cancer mortality in 2018, responsible for >1,000,000 new cases and an estimated 783,000 deaths in the world [1]. Currently available treatments for stomach cancer including surgery, chemotherapy, and radiotherapy are less optimal and the prognosis of stomach cancer is rather poor because of the insufficient understanding of gastric cancer. Thus, it is crucial to explore the molecular carcinogenesis of stomach cancer and develop appropriate treatment, such as target therapy.

Notch signaling is an essential cell-to-cell communication pathway in cell and tissue development [2]. Generally, four Notch receptors (Notch1, Notch2, Notch3, and Notch4) are activated by four ligands [delta-like canonical Notch ligand (DLL)1, DLL4, Jagged1, and Jagged2] and two successive proteolytic cleavages are undergone by metalloproteinases and γ-secretases for the release of the Notch intracellular domain (NICD). The released NICD translocates into the nucleus and binds to the transcription factors of the recombination signal binding protein for the immunoglobulin kappa J region (RBPJ) family. The NICD recruits additional coactivators to induce the transcriptional expression of downstream target genes like hes family bHLH transcription factor 1 (Hes1) [3]. Besides, Notch ligands delta and serrate have also been reported to cell-autonomously antagonize Notch signaling in both vertebrate and invertebrate systems [4–6]. Unlike related Notch ligand family members, DLL3 could not activate Notch signaling, but DLL3 could cell-autonomously inhibit it [7–9]. In addition, DLL3 was highly expressed in many cancers and elevation of DLL3 enhanced the development of many cancers, including small-cell lung cancers [10, 11], pituitary adenomas [12, 13], breast cancer [14, 15], and acute myeloid leukemia [16]. On the contrary, DLL3 has been shown to inhibit hepatocellular carcinoma [17] and glioblastoma [18, 19]. Collectively, activated DLL3 can be oncogenic or tumor-suppressive, depending on the tumor type and cellular context. However, the effect of DLL3 in stomach cancer is barely understood.

Tumor-associated macrophages (TAMs) are one of the main populations of infiltrating immune cells in the tumor microenvironment and are critical mediators of tumor growth [20]. Elevated TAMs were associated with poor outcomes and malignant progression in cancers, such as the suppression of adaptive antitumor immunity and the enhancement of tumor proliferation, migration, invasiveness, and angiogenesis [21–23]. The high density of TAMs was correlated with a poor prognosis in various types of cancers, including stomach cancer [24–27]. CD68^+^ TAMs comprised pro-inflammatory macrophages (M1) and immunosuppressive macrophages (M2) in stomach cancer [28]. Patients with an above-the-median M1/M2 ratio had significantly higher survival rates compared with those with below-the-median ratios [29]. TAMs enrichment characterized by M2-polarized phenotype in stomach cancer could promote the migration of cancer cells [30]. High M2 density was significantly associated with advanced tumor depth, presence of lymph-node metastasis, and shorter cancer-specific survival time [31, 32]. However, the role of TAMs in stomach cancer and the underlying mechanism remain elusive.

Accumulating evidence suggests that TAMs in the tumor microenvironment promote the expressions of various cytokines, which could affect the cancer progression. The intracellular interleukin (IL)-10 and IL-12 status on monocytes in patients with advanced stomach cancer were significantly increased compared with those in patients with early disease or in healthy individuals [33]. Stomach-cancer-derived exosomes can induce PD1^+^ TAM generation and these cells can produce a large amount of IL-10, impair CD8^+^ T-cell function, and create conditions to promote stomach-cancer progression [34]. IL-1β is a major pleiotropic cytokine in tumor progression through effects on angiogenesis, proliferation, invasion, metastases, and myeloid-cell recruitment [35]. IL-1β induced by *Helicobacter pylori* infection enhanced mouse gastric carcinogenesis [36]. IL-1β secreted by tumor-activated monocytes promoted the development of IL-17–producing CD8^+^ T-cell (Tc17) populations and the supernatants induced the production of the chemokine CXCL12 by tumor cells and led to functional impairment of antitumor CD8^+^ cytotoxic T-cells [37]. The exposure of stomach-cancer cells to the medium from lipopolysaccharide (LPS)-treated macrophages significantly induced the production of IL-1β from stomach-cancer cells and promoted cell proliferation [38]. All these results indicate the significant role of IL-1β, IL-12, and IL-10 in the tumor microenvironment in stomach cancer.

It has been reported that Notch signaling regulates the expressions of cytokines and the recruitment of TAMs in breast cancer [39, 40]. However, little is known about how Notch signaling affects TAMs in stomach cancer. In the present study, we demonstrated that the co-culture of macrophages and stomach-cancer cells induced the activation of DLL3-dependent Notch1/Notch2 signaling, the upregulation of IL-33, and the degradation of the galectin-3–binding protein (LG3BP) and heat shock cognate 71 kDa protein (HSPA8), resulting in enhanced proliferation of cancer cells and elevated IL-1β, IL-12, and IL-10 secretion by macrophages.

## Materials and methods

### Cell lines

Human stomach-cancer cell lines (MKN45 and BCG823) and human acute monocytic leukemia cell line THP-1 were from the American Type Culture Collection (ATCC; Manassas, WV, USA) and preserved in our laboratory. All cell lines were cultured in Roswell Park Memorial Institute (RPMI) 1640 medium (Thermo Fisher Scientific; Waltham, MA, USA) with 10% fetal bovine serum (SiJiQing Biotechnology Co.; Hangzhou, Zhejiang, China) and 100 U/mL penicillin and 100 μg/mL streptomycin (Thermo Fisher Scientific). Cells were passaged using 0.05% trypsin-ethylenediaminetetraacetic acid (EDTA; Thermo Fisher Scientific) when cells reached 80%–90% confluency.

#### Co-culture of macrophages and stomach-cancer cells

The polarization of THP-1 into macrophages and the co-culture of macrophages and cancer cells have been illustrated [41]. Briefly, the cells were co-cultured using transwell chambers with a 0.4-μm porous membrane (Corning; New York, NY, USA). THP-1 (1 × 10^5^ cells/mL) were stimulated to differentiate into macrophages by 320 nM phorbol 12-myristate 13-acetate (PMA; Sigma Chemical Co.; St Louis, MO, USA) for 24 h in the upper chamber. The stomach-cancer cells were placed in the lower chamber at a density of 2.5 × 10^5^ cells/mL for 8 h to allow them to adhere to the walls. The chambers with THP-1–derived macrophages were then placed directly on top of the six-well plates containing stomach cells and the co-culture systems were incubated for 6 h in serum-free RPMI 1640 medium. The supernatant was centrifuged at 300 *g* for 5 min and collected.

### DAPT inhibition

Gamma-secretase inhibitor (DAPT; Selleck Chemicals; Houston, TX, USA) was dissolved in 0.08% dimethyl sulfoxide (DMSO) and stored at 4°C. To inhibit Notch signaling, stomach cells were treated with DAPT for 8 h prior to the co-culture of stomach-cancer cells and THP-1–derived macrophages. Then the proteins of stomach-cancer cells were extracted for Western blot. Stomach cells were treated with DMSO as control.

### The overexpression of DLL3 by pCMV-Tag4–DLL3 transfection

Eukaryotic expression plasmid pCMV-Tag4–DLL3 (constructed in our laboratory) was transfected to MKN45 and BCG823 using Lipofectamine 2000 Reagent (Thermo Fisher scientific). The cells were plated in a six-well plate and grew to 70%–80% confluency before transfection with 5 μg pCMV-Tag4–DLL3. Empty vector pCMV-Tag4 was also transfected as control. The ratio of DNA (μg) to Lipofectamine 2000 (μL) is 1:2. After transfection for 48 h, cells were cultured in medium with G418 (Sigma Chemical Co.) for 4 weeks.

### The downregulation of DLL3 by siRNA–DLL3

siRNA–DLL3s were the target-specific 19- to 25-nt siRNAs designed to knock down the human *DLL3* gene. For siRNA transfection, MKN45 and BCG823 cells were seeded into six-well plates to grow to subconfluency and transiently transfected with negative control siRNA (sc-37007; Santa Cruz Biotechnology, Inc.; Santa Cruz, CA, USA) or siRNA–DLL3 (sc-44236; Santa Cruz Biotechnology, Inc.) at 50 nM for 24 h using RNAifectin Transfection Reagent (G073; Applied Biological Materials; Richmond, British Columbia, Canada).

### MTT assay

For cell-proliferation detection, 1 × 10^4^ cells were plated in 96-well plates. After cells had grown for 0 h (30 min after cells were plated), 72 h, and 144 h, 5 mg/mL 3-(4,5-dimethylthiazol-2-yl)-2,5-diphenyltetrazolium bromide (MTT; Sigma Chemical Co.) was added into the medium and cells were cultured at 37°C with 5.0% CO_2_ for 2 h. Then the supernatant was removed carefully and 200 μL DMSO was added to dissolve the precipitation thoroughly. The absorbance of 490 nm was measured using a microplate reader (EPOCH2; BioTek; Winooski, VT, USA).

### ELISA

The concentration of cytokine in the supernatant of the cell culture was quantitative determined using enzyme-linked immunosorbent assay (ELISA), including IL-1β (CSB-E08053h; CUSABIO; Wuhan, Hubei, China), IL-12 (CSB-E04599h; CUSABIO), IL-10 (CSB-E04593h; CUSABIO), and IL-33 (CSB-E13000h; CUSABIO). The results were quantified using a microplate reader (EPOCH2; BioTek).

### Western blot

Cells were collected and lysed using M-PER Mammalian Protein Extraction Reagent (Thermo Fisher scientific). The protein was separated on 10% polyacrylamide gels and transferred electrophoretically to nitrocellulose membranes. After being blocked, the membranes were incubated at 4°C with the following primary antibodies and followed by a secondary antibody at room temperature. Dilution of primary antibodies and secondary antibodies was as follows: Notch1 (1:500; ab27526; Abcam; Eugene, OR, USA), Notch2 (1:1,000; 5732S; Cell signal Technology; Danvers, MA, USA), Notch3 (1:1,000; 5276; Cell signal Technology), Notch4 (1:5,000; 2423; Cell signal Technology), DLL3 (1:1,000; ab103102; Abcam), Hes1 (1:1,000; D6P2U; Cell signal Technology), GAPDH (1:30,000; AP0063; Bioworld Technology; St Louis, MN, USA), Goat anti-Rabbit IgG-HRP (1:10,000; FDR007; Fdbio science; Hangzhou, Zhejiang, China), and Goat anti-Mouse IgG-HRP (1:5,000; FDM007; Fdbio science). The bands of proteins were confirmed by luminescent visualization using an ECL Western Blotting Detection System (Bio-Rad, Hercules, CA, USA) and quantified using the Quantity One software package (Bio-Rad).

### Whole-transcriptome sequencing

DLL3-overexpressed MKN45 (DLL3–MKN45) and MKN45 transfected with empty vector (EV–MKN45) cells were cultured in dishes with a diameter of 10 cm to 80%–90% confluency. We collected the cell lysate using 1 mL RNAiso Plus (Takara; Beijing, China). The cell lysate was stored at −80°C and sent to IGE Biotechnology Company (Guangzhou, Guangdong, China) with dry ice for whole-transcriptome sequencing.

### Co-immunoprecipitation and mass spectrometry

MKN45 cell lysate was diluted with phosphate-buffered saline to roughly 2 μg/μL total cell protein. DLL3 antibody (MAB4315; R&D Systems; Minneapolis, MN, USA) was added to 500 μg cell lysate and the reaction mixture was gently rocked at 4°C overnight. Then, 100 μL (50 μL packed beads) of washed protein A agarose and 20 μL (10 μL packed beads) of washed protein G agarose (Millipore; Burlington, NJ, USA) were added to the mixture to capture the immunocomplex with gentle rocking at 4°C for 2 h. The agarose beads were collected by pulsing (5 s at 14,000 rpm). The beads were washed three times using ice-cold cell lysis buffer and resuspended in 60 μL 2× Laemmli sample buffer (Thermo Fisher scientific). The beads were boiled for 5 min and the supernatant was collected using a microcentrifuge pulse for Western blot. After immunoprecipitation with the antibody of DLL3 in MKN45 lysate, the sample was analysed using mass spectrometry in the Beijing Proteome Research Center (Beijing, China). The proteins identified were excluded if the unused score was <1.3 and peptides (95%) <2 based on ProteinPilot Software 5.0.2 (AB Sciex; Framingham, MA, USA). The common pollutants and proteins in the immunoglobulin G (IgG) control group were also excluded.

### Gene-based analysis via the UCSC

Gene analysis in stomach cancer was performed in University of California, Santa Cruz (UCSC) Xena Functional Genomics Explorer (https://xenabrowser.net/heatmap/). The study selected was The Cancer Genome Atlas (TCGA) Stomach Cancer. The first valuable selected was Phenotype-sample type, and the second valuable selected was Genomic—Gene name—Gene expression RNA-seq (IlluminaHiSeq pancan normalized) plus Exon expression RNA-seq (IlluminaHiSeq UNC). All the null data should be removed using the filter ‘! = null’. About the Kaplan–Meier plot, the filter ‘primary’ should be added when comparing the survival rate between patients with high and low target genes expression. The gene-expression level in the Kaplan–Meier plot is white < blue < red or gray < blue < red.

### Tissue specimens and immunohistochemistry

The present study was approved by the Protection of Human Subjects Committee of Southern Medical University (Guangzhou, Guangdong, China). A total of 35 tissue samples from patients with stomach cancer were collected and all patients signed an informed consent statement and agreed to the use of their tissues. The immunohistochemistry was performed by Linked-biotech Pathology Co. Ltd (Guangzhou, Guangdong, China). The slides were graded using semi-quantitative analysis based on the staining-intensity scoring (0 for no staining; 1 for faint yellow; 2 for clay bank; 3 for tan) and the positive-staining-percentage scoring of target cells (0 for 0%–5%; 1 for 6%–25%; 2 for 26%–50%; 3 for 51%–75%; 4 for >75%). Then the slides were categorized into four groups according to the product of the staining-intensity score and the positive-staining-percentage score as follows: 0 for negative (−), 1–4 for weakly positive (+), 5–8 for moderately positive (++), and 9–12 for strongly positive (+++).

### Statistical analysis

Statistical analysis was performed using GraphPad 6.0 software (GraphPad Software; San Diego, CA, USA). Data are shown as mean ± standard deviation (SD). The Student’s *t*-test was used to analyse the differences between the two groups. Welch’s correction was applied when the variances of the two compared groups were not equal. Differences were considered significant when the *P-*value was <0.05. ^*^P < 0.05, ^**^P < 0.01, ^***^P < 0.001.

## Results

### The co-culture of stomach-cancer cells and macrophages activated Notch1/Notch2 signaling, upregulated DLL3, and promoted cancer-cell proliferation

**Figure 1.**
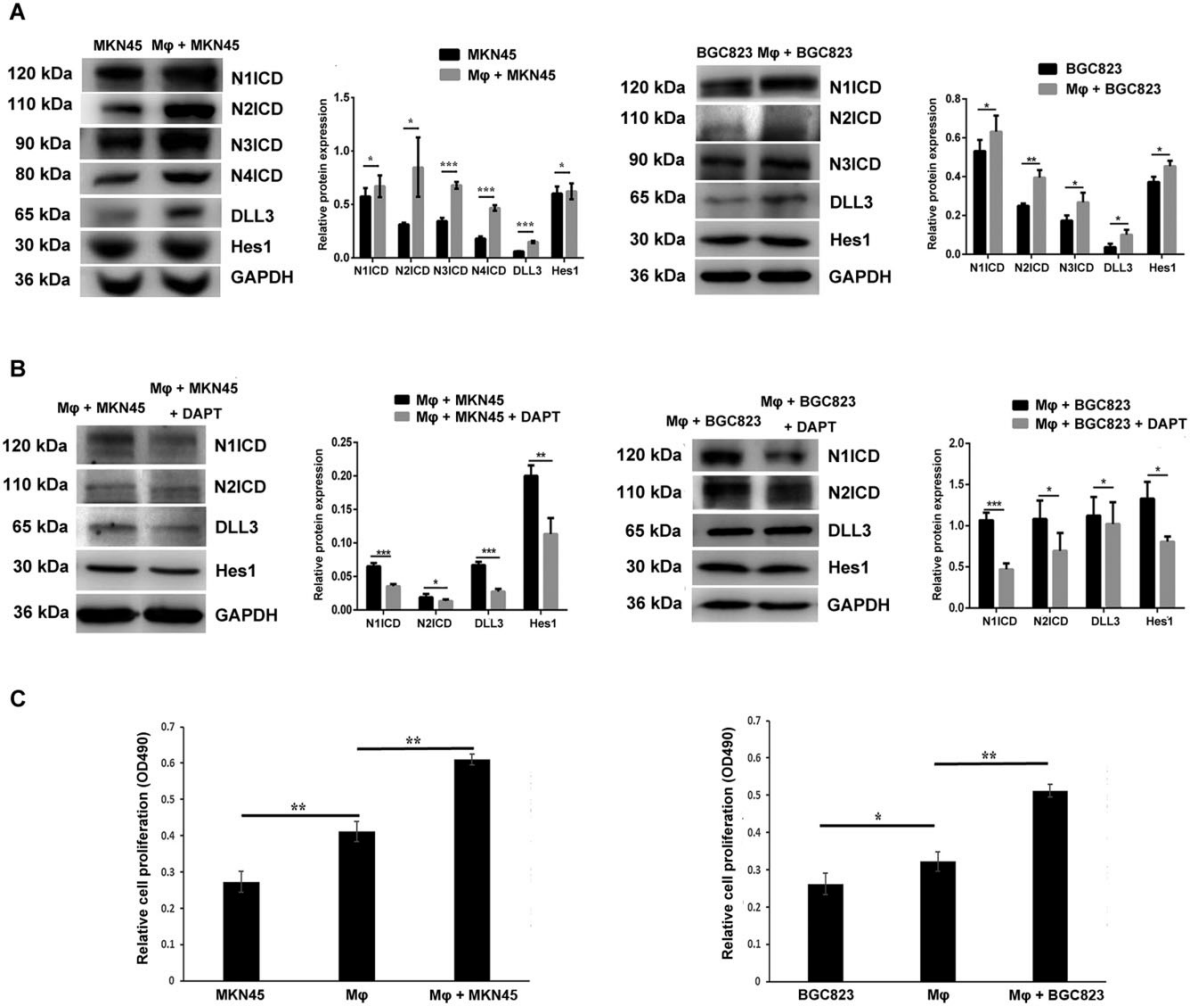
The changes in Notch signal proteins in stomach-cancer cells and cell proliferation after co-culture with macrophages. (A) The expressions of N1ICD, N2ICD, N3ICD, N4ICD, DLL3, and Hes1 in MKN45 and BGC823 were detected by Western blot after co-culture with macrophages (Mφ) for 6 h. Each histogram indicates the relative band intensity compared with internal control GAPDH. (B) The changes in Notch signal proteins in MKN45 and BGC823 were detected after cells were treated with DAPT for 8 h prior to co-culture with macrophages. Each histogram indicates the relative band intensity compared with internal control GAPDH. (C) The proliferation of MKN45 and BGC823 cultured by supernatants from MKN45, BGC823, macrophages, and co-cultures (Mφ + MKN45, Mφ + BGC823) for 24 h was detected by MTT assay. All these experiments were repeated three times in triplicate. Statistical significance is indicated by ^*^P < 0.05, ^**^P < 0.01, and ^***^P < 0.001. N1ICD, Notch1 intracellular domain; N2ICD, Notch2 intracellular domain; N3ICD, Notch3 intracellular domain; N4ICD, Notch4 intracellular domain; DLL3, delta-like ligand 3; Hes1, hes family bHLH transcription factor 1; GAPDH, glyceraldehyde 3-phosphate dehydrogenase; DAPT, Notch signaling gamma-secretase inhibitor; MTT, 3-(4,5-dimethylthiazol-2-yl)-2,5-diphenyltetrazolium bromide.

To understand how macrophages affect stomach cancer, we established an *in vitro* co-culture model of stomach-cancer cells and THP-1–derived macrophages. The protein expressions of Hes1, DLL3, and activated forms of four Notch receptors (N1ICD, N2ICD, N3ICD, and N4ICD) were all upregulated in co-culture groups (Figure 1A). To inhibit Notch signaling, stomach-cancer cells were treated with DAPT prior to the co-culture with macrophages. The protein-expression levels of N1ICD, N2ICD, DLL3, and Hes1 were downregulated in the DAPT-treated co-culture group compared with those in the co-culture group without DAPT (Figure 1B). These results demonstrated that Notch signaling was activated comprehensively in stomach cancer after co-culture with macrophages.

Since many studies have proved the oncogenic effect of Notch1 and Notch2, we collected the supernatant from the co-culture as the cell-culture-conditioned medium and measured cell proliferation using MTT assay to verify the effect of co-culture on the proliferation of cancer cells. Cancer cells grew faster in the supernatant from macrophages than those in the supernatant from cancer cells, and cancer cells grew even faster in the supernatant from co-culture (Figure 1C). These results suggested that the co-culture of macrophages and MKN45 could enhance cell proliferation through activating DLL3–Notch1/Notch2 signaling.

### DLL3 promoted stomach-cancer-cell proliferation, migration, and cell cycle

**Figure 2.**
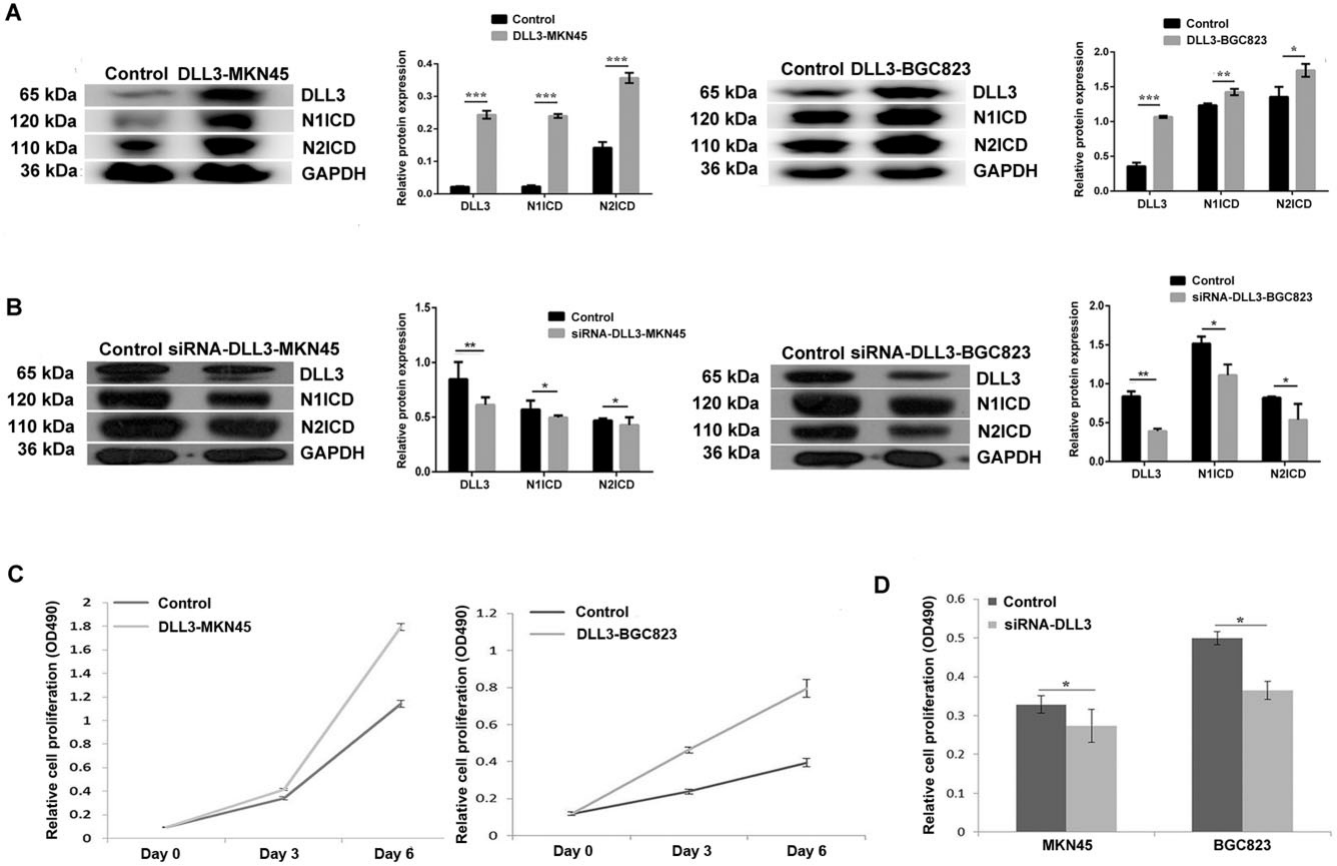
The changes in Notch signal proteins in stomach-cancer cells and cell proliferation after the overexpression and downregulation of DLL3. (A) The expressions of N1ICD, N2ICD, and DLL3 were detected by Western blot after MKN45 and BGC823 cells were transfected with pCMV-Tag4–DLL3. In controls, cells were transfected with empty vector pCMV-Tag4. Each histogram indicates the relative band intensity compared with internal control GAPDH. (B) The expressions of N1ICD, N2ICD, and DLL3 were detected by Western blot after DLL3 was downregulated by siRNA in MKN45 and BGC823 cells. Cells were transfected with control siRNA in control groups. Each histogram indicates the relative band intensity compared with internal control GAPDH. (C) The proliferation of MKN45 and BGC823 with DLL3 overexpression was detected by MTT on day 3 and day 6. Cells were transfected with empty vector pCMV-Tag4 in controls. Data are represented as mean ± SD of three experiments. (D) The proliferation of MKN45 and BGC823 with DLL3 downregulation was detected by MTT after siRNA–DLL3 was transfected for 48 h. In controls, cells were transfected with control siRNA. Data are represented as mean ± SD of three experiments. Statistical significance is indicated by ^*^P < 0.05, ^**^P < 0.01, and ^***^P < 0.001. DLL3, delta-like ligand 3; N1ICD, Notch1 intracellular domain; N2ICD, Notch2 intracellular domain; GAPDH, glyceraldehyde 3-phosphate dehydrogenase; SD, standard deviation; MTT, 3-(4,5-dimethylthiazol-2-yl)-2,5-diphenyltetrazolium bromide.

DLL3 was overexpressed or inhibited in MKN45 and BGC823 to further investigate the oncogenic effect of DLL3 on stomach cancer. In DLL3-overexpressed MKN45 (DLL3–MKN45) and BGC823 (DLL3–BGC823) cells, the protein-expression levels of N1ICD and N2ICD were upregulated (Figure 2A). In contrast, those were downregulated in siRNA-mediated DLL3-inhibited MKN45 (siRNA–DLL3–MKN45) and BGC823 (siRNA–DLL3–BGC823) cells (Figure 2B). DLL3-overexpressed MKN45 and BGC823 proliferated faster than corresponding control cells on day 3 and day 6 (Figure 2C). On the contrary, DLL3–downregulated cells proliferated at a lower rate in comparison with controls (Figure 2D). In addition, migration was enhanced in DLL3-overexpressed MKN45 and BGC823 cells (Supplementary Figure 1). For the cell cycle, stably DLL3-overexpressed cells were first detected by flow cytometry using Annexin V fluorescein isothiocyanate/propidium iodide (Annexin V FITC/PI) staining to distinguish the state of apoptosis. The results showed that there was no significant difference between the treated cells and the control, suggesting that DLL3 may not work through apoptosis signaling. Downregulation of DLL3 increased the percentage of cells in the G0/G1 phase at the expense of the S phase in MKN45 and BGC823 (Supplementary Figure 2), suggesting that DLL3 downregulation decreased G1/S transition in gastric-cancer cells. Collectively, these data indicated that DLL3 promoted cell proliferation and migration in stomach-cancer cells.

### The overexpression of DLL3 in stomach-cancer cells increased secretion of IL-10 and IL-12 by macrophages

**Figure 3.**
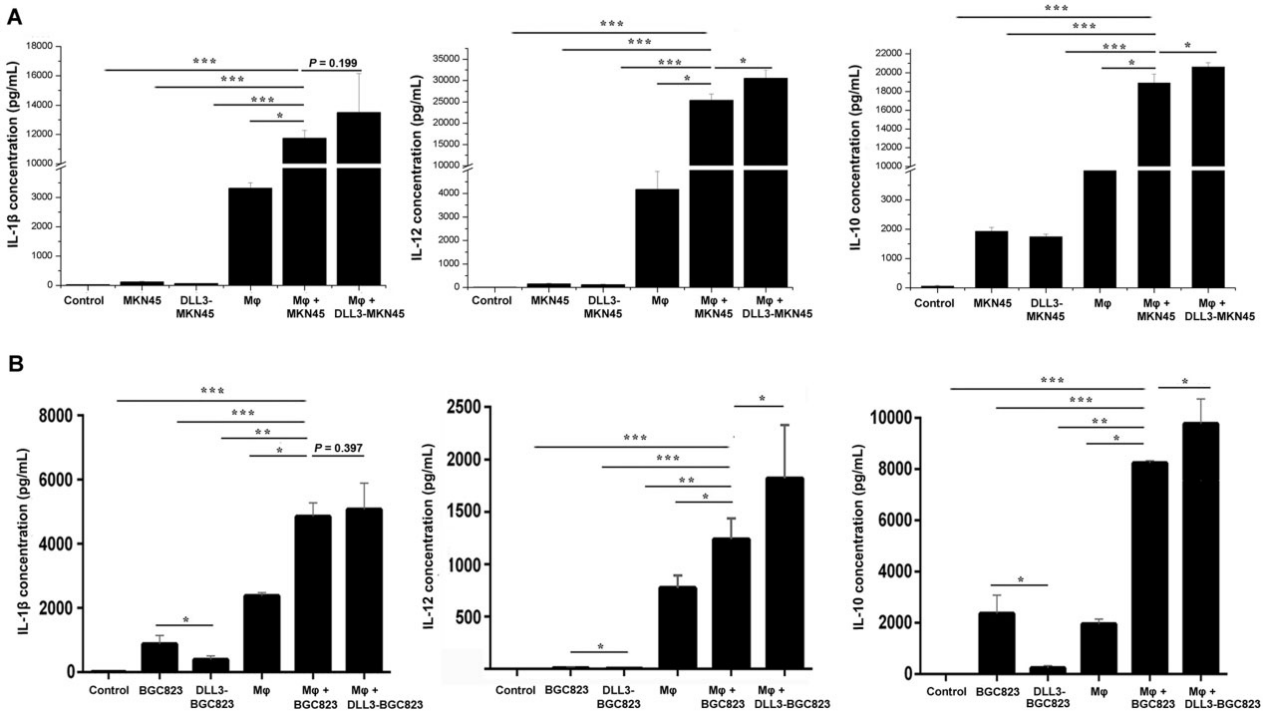
The expressions of IL-1β, IL-12, and IL-10 in the culture supernatant were detected by ELISA after co-culture of macrophages and stomach-cancer cells. Control, RPMI 1640 medium; MKN45/BGC823, supernatant from MKN45/BGC823 cells; DLL3–MKN45/DLL3–BGC823, supernatant from DLL3-overexpressed MKN45/BGC823 cells; Mφ, supernatant from macrophages; Mφ + MKN45/Mφ + BGC823, supernatant from co-culture of macrophages and MKN45/BGC823 cells; Mφ + DLL3–MKN45/Mφ + DLL3–BGC823, supernatant from co-culture of macrophages and DLL3-overexpressed MKN45/BGC823 cells. Error bars represent standard error of means from three independent experiments. Statistical significance is indicated by ^*^P < 0.05, ^**^P < 0.01, and ^***^P < 0.001. IL, interleukin; DLL3, delta-like ligand 3; ELISA, enzyme-linked immunosorbent assay.

To explore whether DLL3 in stomach cancer affects cytokine secretion by macrophages, macrophages were co-cultured with cancer cells or DLL3-overexpressed cancer cells. The supernatants from the culture medium of cancer-cell groups (MKN45, BGC823), DLL3-overexpressed-cancer-cell groups (DLL3–MKN45, DLL3–BGC823), macrophage (Mφ) groups, macrophage-with-cancer-cell groups (Mφ + MKN45, Mφ + BGC823), and macrophage-with-DLL3-overexpressed-cancer-cell groups (Mφ + DLL3–MKN45, Mφ + DLL3–BGC823) were collected and the expression levels of IL-1β, IL-12, and IL-10 were detected using ELISA. The RPMI 1640 was used as a blank control. As shown in Figure 3, IL-1β, IL-12, and IL-10 were all upregulated in the supernatants from the macrophage-with-cancer-cell groups compared with those from the control, cancer-cell, DLL3-overexpressed-cancer-cell, and macrophage groups. IL-12 and IL-10 were further upregulated in the supernatant from the macrophage-with-DLL3-overexpressed-cancer-cell groups compared with those from the macrophage-with-cancer-cell groups, but there was no significant difference for IL-1β between these two groups. To be noticed, the secretions of IL-1β, IL-12, and IL-10 were decreased in the DLL3-overexpressed-cancer-cell groups compared with those in the cancer-cell groups. These results indicated that the increased secretion of IL-1β, IL-12, and IL-10 was from macrophages in the co-culture of macrophages and DLL3-overexpressed cancer cells. Since the co-culture was performed by transwell and DLL3-overexpressed cancer cells had no direct contact with macrophages, DLL3 overexpression in cancer cells affected macrophages via a unique mechanism.

### The overexpression of DLL3 in MKN45 cells induced upregulation of IL-33

**Figure 4.**
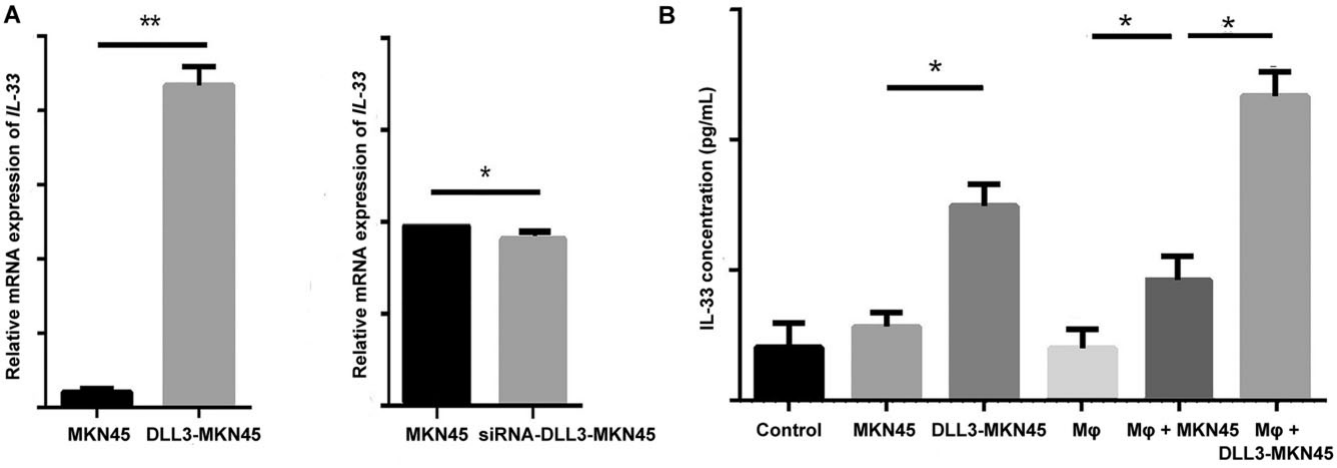
The expression of IL-33 in DLL3-overexpressed MKN45 cells. (A) The mRNA expression of *IL-33* in DLL3-overexpressed and DLL3–downregulated MKN45 cells was detected by qPCR. The relative expression level of mRNA was normalized to β-actin expression. MKN45, MKN45 cells; DLL3–MKN45, DLL3-overexpressed MKN45 cells; siRNA–DLL3–MKN45, DLL3-downregulated MKN45 cells. (B) The expression of IL-33 in the supernatant was detected by ELISA after co-culture of macrophages and DLL3-overexpressed MKN45 cells. Control, RPMI 1640 medium; MKN45, supernatant from MKN45 cells; DLL3–MKN45, supernatant from DLL3-overexpressed MKN45 cells; Mφ, supernatant from macrophages; Mφ + MKN45, supernatant from co-culture of macrophages and MKN45; Mφ + DLL3–MKN45, supernatant from co-culture of macrophages and DLL3-overexpressed MKN45 cells. Error bars represent standard error of means from three independent experiments. Statistical significance is indicated by ^*^P < 0.05 and ^**^P < 0.01. IL, interleukin; DLL3, delta-like ligand 3; ELISA, enzyme-linked immunosorbent assay.

Human total transcriptome sequencing of DLL3–MKN45 mRNA was performed to further investigate whether DLL3 overexpression in MKN45 affects macrophages. The genes with a significant change (*P* < 0.01, log2-fold change >2) in mRNA levels are displayed in Table 1, and *DLL3*, nuclear receptor corepressor 1 pseudogene 3 (*NCOR1P3*), and *IL-33* of them were related to macrophages. The details of NCOR1P3 could not be retrieved since it is a pseudogene. IL-33 has been proved to promote colon-cancer-cell stemness via the c-Jun N-terminal kinase (JNK) activation and macrophage recruitment [42]. Furthermore, the mRNA expression of *IL-33* in DLL3–MKN45 was higher than that in MKN45 by quantitative real-time polymerase chain reaction. In contrast, the siRNA-mediated knock-down of DLL3 in MKN45 decreased *IL-33* expression (Figure 4A). Additionally, MKN45 could secret more IL-33 after co-culture with macrophages and DLL3–MKN45 even induced higher secretion of IL-33 after co-culture with macrophages than MKN45 with macrophages did (Figure 4B). Thus, the above results demonstrated that the overexpression of DLL3 in MKN45 could result in the upregulation of IL-33 and this induction was enhanced via co-culture with macrophages.

**Table 1.** Genes regulated by DLL3 overexpression in MKN45

Gene name	Official full name	Fold change
*NID1*	nidogen 1	−4.36
*MIR34AHG*	MIR34A host gene	−4.14
*ZNF705E*	zinc finger protein 705E	−2.78
*RP11-570J4.1*	n/a	−2.55
*RP11-370K11.1*	n/a	−2.46
*SLC38A5*	solute carrier family 38 member 5	−2.42
*CCDC65*	coiled-coil domain containing 65	−2.37
*ROBO1*	roundabout guidance receptor 1	−2.37
*DLL3*	delta-like canonical Notch ligand 3	4.36
*IL-33*	interleukin 33	2.90
*CXADRP1*	Ig-like cell adhesion molecule pseudogene 1	2.79
*NCOR1P3*	nuclear receptor corepressor 1 pseudogene 3	2.23
*ZNF595*	zinc finger protein 595	2.22
*RP11-377G16.2*	n/a	2.32
*ACTG2*	actin, gamma 2, smooth muscle, enteric	2.04

Underline indicates genes related to macrophages.

### IL-33 promoted the secretion of IL-10 and IL-12 by macrophages

**Figure 5.**
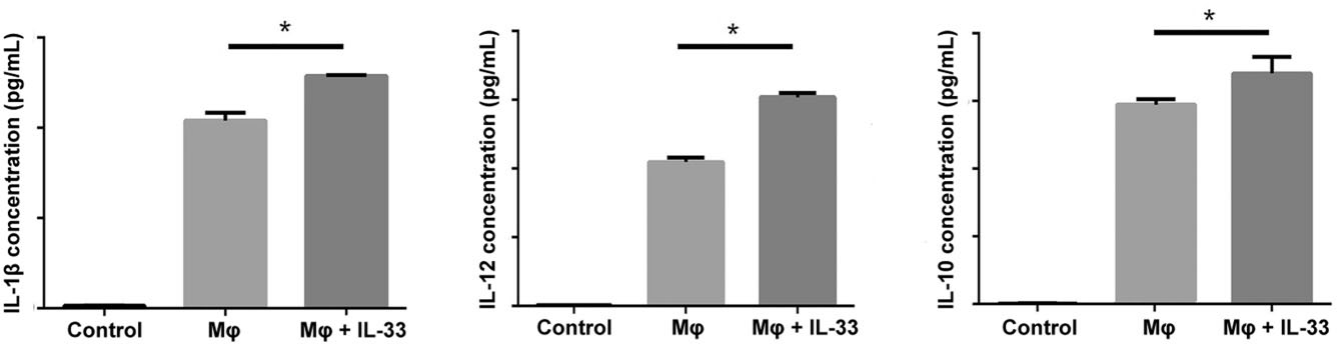
The expressions of IL-10, IL-1β, and IL-12 after macrophages were stimulated by IL-33 for 48 h. Control, RPMI 1640 medium; Mφ, supernatant from macrophages; Mφ + IL-33, supernatant from macrophages stimulated with interleukin (IL)-33. Error bars represent standard error of means from three independent experiments. Statistical significance is indicated by ^*^P < 0.05.

To further explore whether upregulation of IL-33 in stomach-cancer cells (MKN45 and BGC823) affects the secretion of IL-1β, IL-12, and IL-10 by the macrophages, ELISA was applied to detect the expressions of IL-1β, IL-12, and IL-10 in the supernatant after macrophages were stimulated with IL-33. The results showed that IL-33 could induce secretion of IL-1β, IL-12, and IL-10 by macrophages (Figure 5).

### DLL3 may induce the degradation of LG3BP and HSPA8

**Figure 6.**
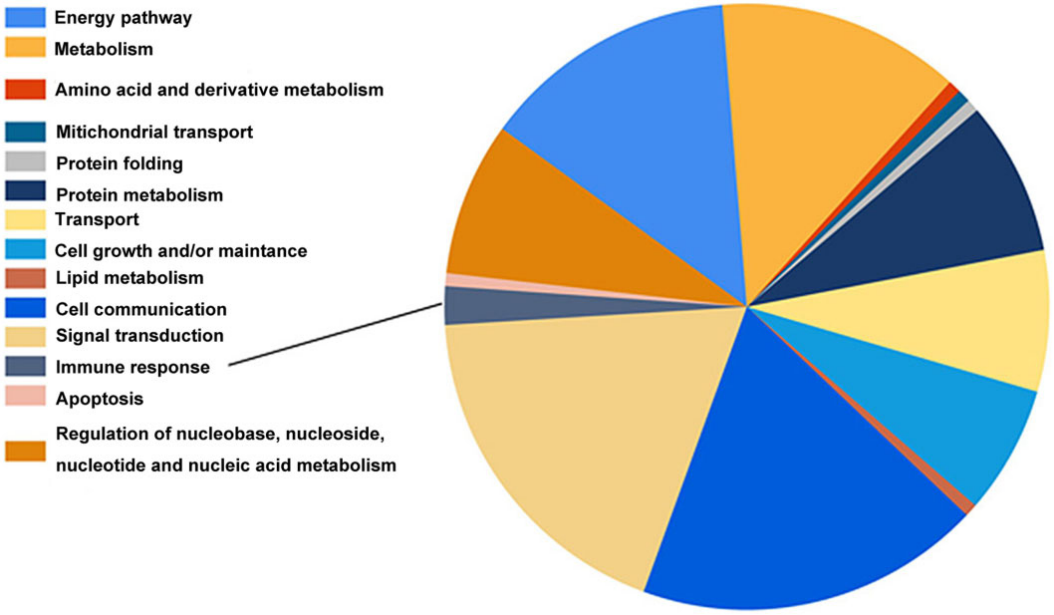
Biological process enrichment of DLL3-interacted proteins identified by co-immunoprecipitation

Co-immunoprecipitation and mass spectrometry (IP-MS) were performed to detect the proteins that interacted with DLL3 in MKN45. After background correction, 218 proteins were identified. The proteins were analysed using FunRich_3.1.3 software (http://www.funrich.org/index.html) [43]. Gene-ontology enrichment analysis of the biological process showed that five proteins that interacted with DLL3 were immune response-related (Figure 6), including human leukocyte antigen class I histocompatibility antigen, C (1C03), histocompatibility minor 13 (HM13), complement decay-accelerating factor, also CD55 (DAF), LG3BP, and HSPA8. Only LG3BP [44] and HSPA8 [45] have been reported as immunostimulator in macrophages or monocytes. Since DLL3 could target newly synthesized Notch1 or DLL1 for endosomal/lysosomal degradation prior to post-translational processing and cell-surface presentation of receptors [8, 9], a convincing explanation for the interaction of DLL3 with LG3BP and HSPA8 is that DLL3 could target LG3BP and HSPA8 for endosomal/lysosomal degradation to inhibit their immunostimulatory function.

### DLL3 or IL-33 was associated with a lower survival rate in patients with stomach cancer

**Figure 7.**
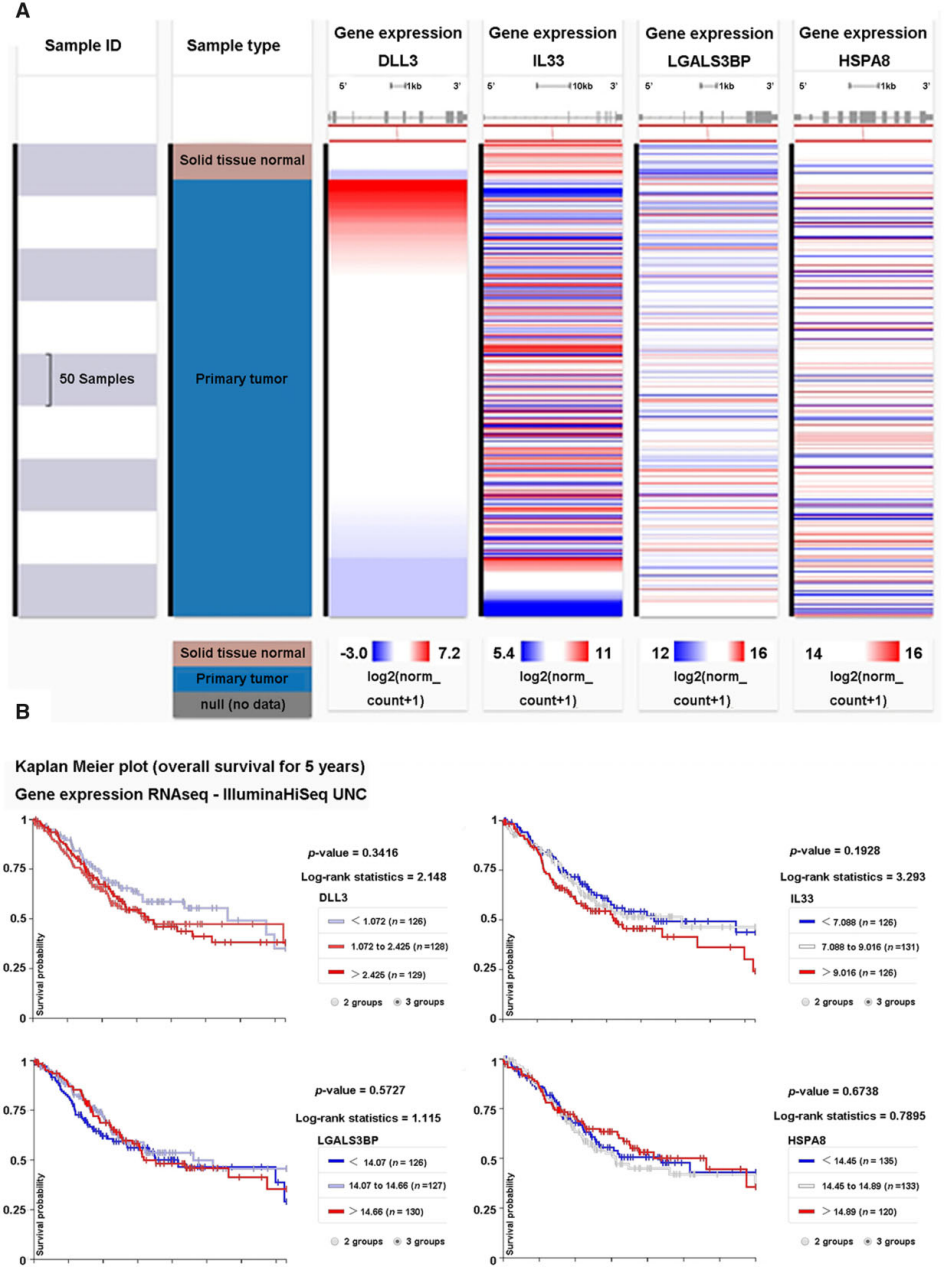
The gene expressions of *DLL3*, *IL-33*, *LG3BP* (also *LGASLS3BP*), and *HSPA8*, and their associations with overall survival were analysed using a UCSC Xena Functional Genomics Explorer. (A) Heat maps of *DLL3*, *IL-33*, *LG3BP*, and *HSPA8* expressions in patients with stomach cancer and normal persons. This data set shows the gene-level transcription estimates, as in log2(*x* + 1) transformed RSEM normalized count. The default view was set to center each gene or exon to zero by independently subtracting the mean of each gene or exon on the fly. (B) Kaplan–Meier plot is displayed to compare the 5-year survival rate between patients with high target gene expression and low expression. *DLL3*, delta-like ligand 3; *IL-33*, interleukin-33; *LG3BP*, galectin-3–binding protein; *HSPA8*, heat shock cognate 71 kDa protein; RSEM, RNA-seq by expectation maximization.

**Figure 8.**
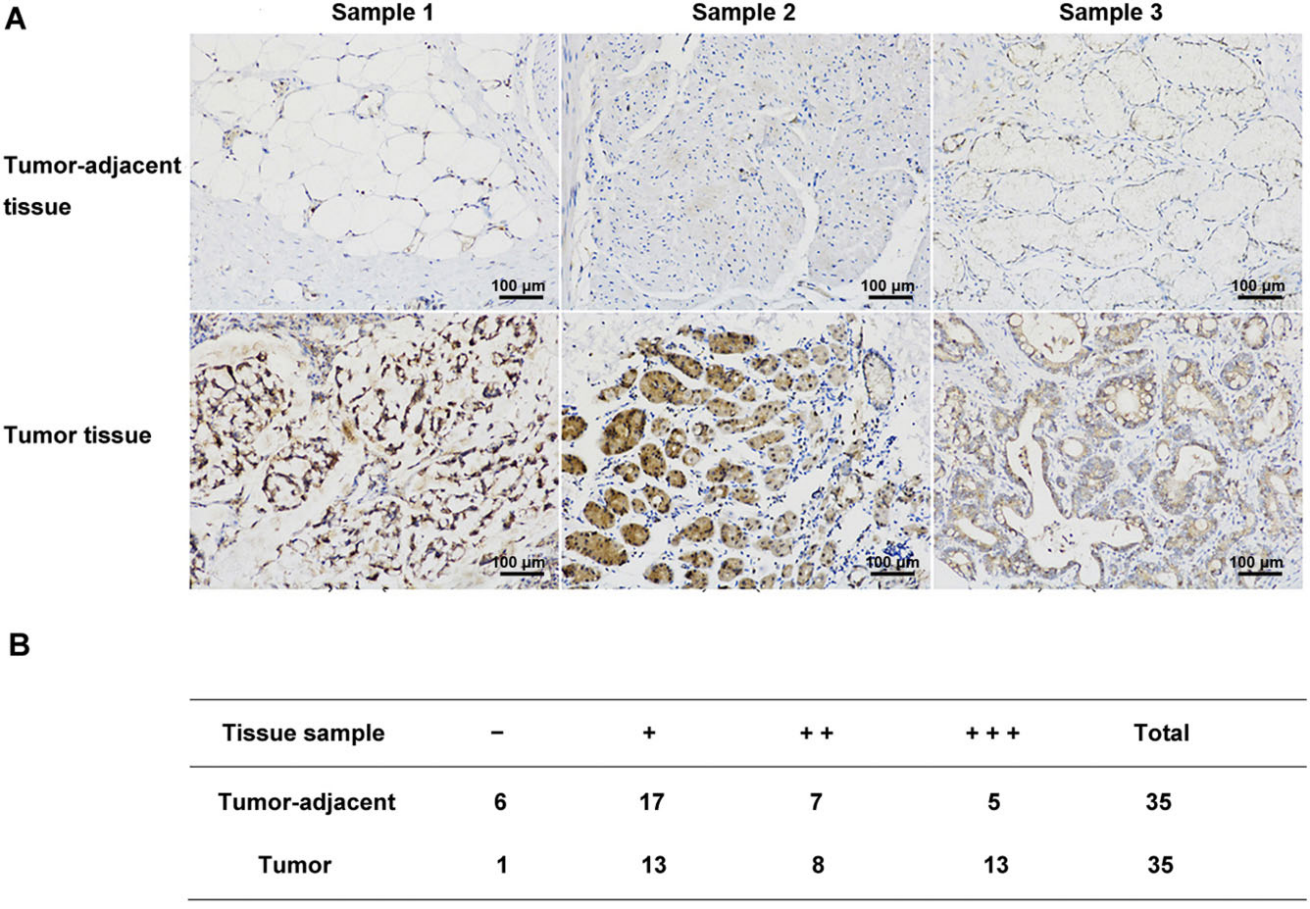
The expression of DLL3 in stomach-cancer tissue. (A) The expression of delta-like ligand 3 (DLL3) in stomach-cancer and corresponding tumor-adjacent tissues was detected by immunohistochemistry using DLL3 antibody. Three pairs of representative samples are displayed. (B) The expression levels of 35 stomach-cancer tissues were categorized into negative (−), weakly positive (+), moderately positive (++), and strongly positive (+++). Scale bar = 100 µm (200× magnification).

Moreover, gene expressions of *DLL3*, *IL-33*, *LG3BP* (also *LGASLS3BP*), and *HSPA8* were analysed in UCSC Xena Functional Genomics Explorer based on the TCGA data set from 415 primary tumor samples of stomach cancer and 35 normal tissues (Figure 7A). The expression of DLL3 was elevated in parts of tumor samples (red), with low expression in most normal tissues (white or blue). The expression of IL-33 was high in normal tissues but low in parts of tumor samples. The expressions of LG3BP and HSPA8 were both low in normal tissues but high in parts of tumor samples. Then, the 5-year overall survival curves were analysed using the Kaplan–Meier method and log-rank test. The result revealed that higher expressions of either DLL3 or IL-33 were associated with a lower survival rate, though without significant differences (Figure 7B). Furthermore, we collected 35 stomach-cancer tissues and corresponding tumor-adjacent tissues to assess DLL3 expression by immunohistochemistry (Figure 8). The expression of DLL3 in tissues was classified as follows: negative (−), weakly positive (+), moderately positive (++), and strongly positive (+++). The protein level of DLL3 was significantly higher (++/+++) in gastric-cancer tissues (21/35) in comparison with that in tumor-adjacent tissues (12/35). These data suggested that the protein expression of DLL3 was elevated in some patients with stomach cancer.

## Discussion

**Figure 9.**
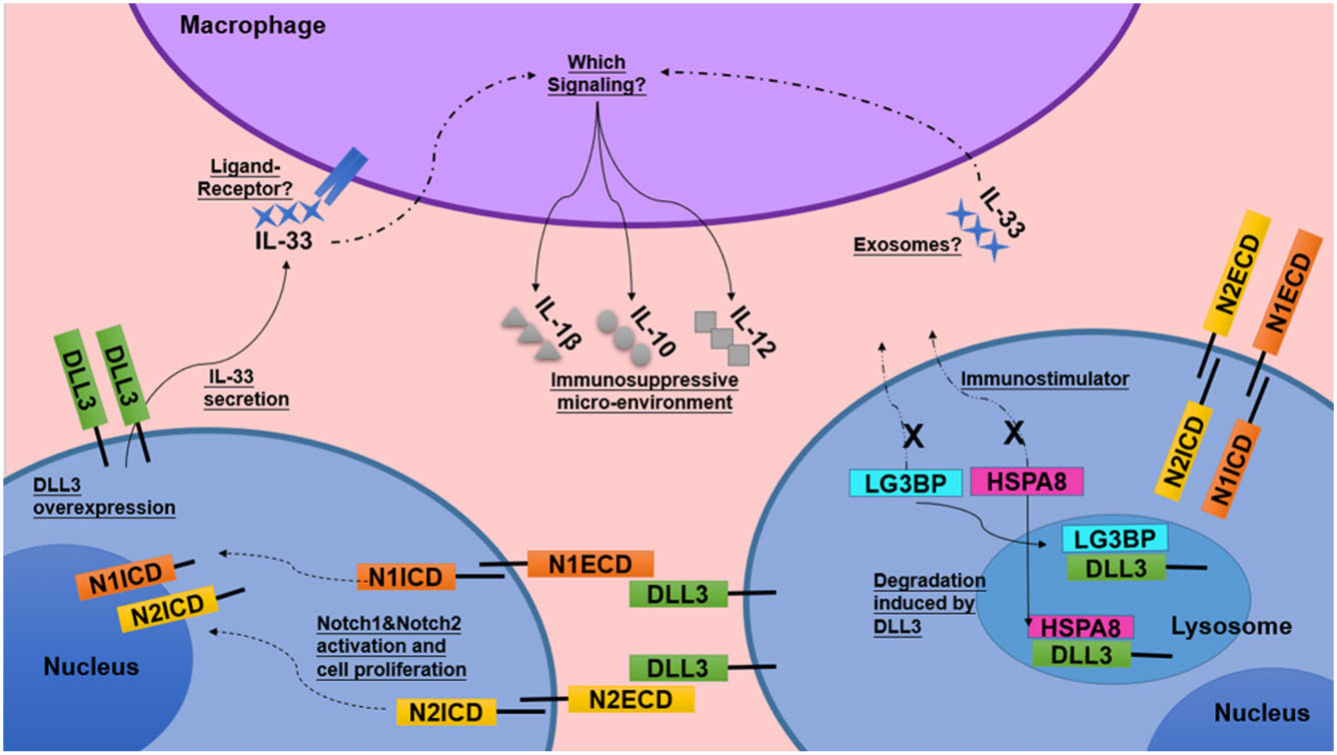
The affection of elevated DLL3 from stomach-cancer cells on both cancer cells and tumor-associated macrophages. DLL3, delta-like ligand 3; N1ECD, Notch1 extracellular domain; N1ICD, Notch1 intracellular domain; N2ECD, Notch2 extracellular domain; N2ICD, Notch2 intracellular domain; LG3BP, galectin-3-binding protein; HSPA8, heat shock cognate 71 kDa protein; IL, interleukin.

The current understanding is that the Notch signal pathway, especially Notch1/Notch2 signaling, has an oncogenic effect on stomach cancer [46–50]. Increasing studies have demonstrated that Notch signaling regulates not only the pre-malignant or malignant cells, but also non-malignant cells such as TAMs in the tumor microenvironment [39, 51, 52]. Like Jagged 1, one of the Notch signaling ligands could drive cytokine expression in TAMs through Notch signaling in breast cancer [38]. To date, whether Notch signaling affects TAMs in stomach cancer is barely understood. Though the co-culture of macrophages and stomach-cancer cells (MKN45 and BGC823) and the detection of related proteins expression and cell activities, we showed that the Notch ligand DLL3 could regulate stomach-cancer-cell proliferation and the cytokine secretion of stomach-cancer cells and macrophages. Our data suggested that Notch signaling was activated in stomach cancer by macrophages. This activation enhanced the proliferation and migration of cancer cells, and facilitated the expression of IL-33 cytokine by DLL3 and the degradation of LG3BP and HSPA8. The release of IL-33 and degradation of LG3BP and HSPA8 increased IL-1β, IL-12, and IL-10 secretion by macrophages (Figure 9).

DLL3 is well known for its effects in somitogenesis and neurogenesis [53–55]. DLL3 also functions in hematopoietic development [56] and the positive selection of autoreactive T-cells [57]. Moreover, elevated DLL3 could exacerbate the progression of many cancers including small-cell lung cancer [10, 11] and pituitary adenoma [12]. These findings promote the development of DLL3-targeted antibody-drug conjugate (ADC) [58] with demonstrated efficacy in preclinical [59] and in phase I clinical trials towards small-cell lung cancer [11]. Recently, treatment of DLL3-expressing prostate-cancer xenografts with this ADC resulted in complete and durable responses, whereas DLL3-negative models were insensitive [60]. On the contrary, DLL3 has been shown to inhibit hepatocellular carcinoma [17] and glioblastoma [18, 19]. Collectively, activated DLL3 can be oncogenic or tumor-suppressive, depending on the tumor type and cellular context. Therefore, the opposite effect of DLL3 on cancers suggests the necessity to check the activation patterns and potential roles of DLL3 in different tumor types without any initial impression. Our data may shed light on the oncogenic effect of DLL3 on stomach cancer. The upregulation of DLL3 in stomach cancer could be induced by macrophages. Based on our data, activated forms of Notch1, Notch2, Notch3, and Notch4 (N1ICD, N2ICD, N3ICD, and N4ICD) in stomach-cancer cells were all upregulated, especially N1ICD and N2ICD, after the co-culture of cancer cells and macrophages.

Information from clinical samples has proved that high expressions of IL-1β, IL-12, and IL-10 in macrophages or in plasma indicate poor progression of stomach cancer [33, 34, 36, 37]. Although IL-1β is known as a pro-inflammatory cytokine, it has been proved that it enhances stomach carcinogenesis in several studies [36–38, 61]. However, whether stomach cancer regulates the cytokine secretion of macrophages is not completely understood. Wang *et al.* demonstrated that stomach-cancer-derived exosomes could educate monocytes to differentiate into PD1^+^ TAMs, which produced a large amount of IL-10, impaired CD8^+^ T-cell function, and thereby created conditions to promote stomach-cancer progression [34]. Our study illustrated that DLL3 in stomach cancer could induce IL-1β, IL-12, and IL-10 secretion of macrophages via up-regulating IL-33. The expression of *IL-33* mRNA in DLL3–MKN45 was higher than that in MKN45. Additionally, DLL3–MKN45 even secreted more IL-33 after being co-cultured with macrophages than MKN45, suggesting that the overexpression of DLL3 in MKN45 could induce upregulation of IL-33 and this induction was enhanced via co-culturing with macrophages. Previous studies demonstrated that IL-33 in colon cancer could activate core stem-cell genes and induce the phosphorylation of JNK activation and enhanced binding of c-Jun to the promoters of core stem-cell genes. IL-33 recruited macrophages into the cancer microenvironment and stimulated them to produce prostaglandin E2, which supported colon-cancer stemness and tumor growth [42]. Together, these experiments indicate a paracrine-positive feedback circuit in cancer in which TAMs induce tumor-cell Notch-dependent secretion of cytokines, and this induction may also take effect through the degradation of LG3BP and HSPA8. LG3BP has been reported to possess an immunostimulatory function in macrophages [44]. HSPA8 could bind bacterial LPS and mediate LPS-induced inflammatory response, including tumor necrosis factor secretion by monocytes. These four proteins, including DLL3, IL-33, LG3BP, and HSPA8, could be a new insight into and promising diagnostic or therapeutic targets of stomach cancer.

Though we have offered a new insight into the effect of Notch signaling between stomach-cancer cells and macrophages, more evidence could be elaborated on to support this idea. A xenograft model could be established to testify the effect of DLL3 *in vivo*. The changes in LG3BP and HSPA8 should be further confirmed by Western blot and ELISA.

In summary, macrophages could induce the activation of DLL3-dependent Notch1/Notch2 signaling, the upregulation of IL-33, and the degradation LG3BP and HSPA8, resulting in enhanced cancer-cell proliferation and elevated IL-1β, IL-12, and IL-10 secretion by macrophages. This is a new insight into the correlation between stomach cancer and macrophages connected by the Notch signaling pathway. Therefore, potential therapy may depend on the downregulation of the DLL3–Notch pathway to suppress the protumorigenic functions of TAMs in stomach cancer.

## Supplementary Data

Supplementary data is available at *Gastroenterology Report* online.

## Authors’ Contributions

J.B.Y. contributed, designed the majority of the work, and wrote this manuscript. J.J.W. and D.L.W. performed experiments, analysed data, wrote the draft, and reviewed the manuscript. B.X.H., M.Q.L., and Y.Q.L. performed some of the experiments and analysed data. Y.L. and Y.S.N. designed the project and wrote the manuscript. All authors read and approved the final manuscript.

## Funding

This work was supported by the National Natural Science Foundation of China [81971903 to Y.L.], the National Key R&D Program of China, Synthetic Biology Research [2019YFA0903802 to Y.N.], and the Innovative Experiment Program of College Students of Guangdong Province, China [No. S202012121166, 202112121330, 202112121335].

## Conflict of Interest

None declared.

## Supplementary Material

goab052_Supplementary_Data
