# Supplementary figures and images for: Elevated DLL3 in stomach cancer by tumor-associated macrophages enhances cancer-cell proliferation and cytokine secretion of macrophages
Source: Gastroenterol Rep (Oxf). 2021 Nov 25;10:goab052. doi: 10.1093/gastro/goab052 (PMC8973010; doi:10.1093/gastro/goab052)

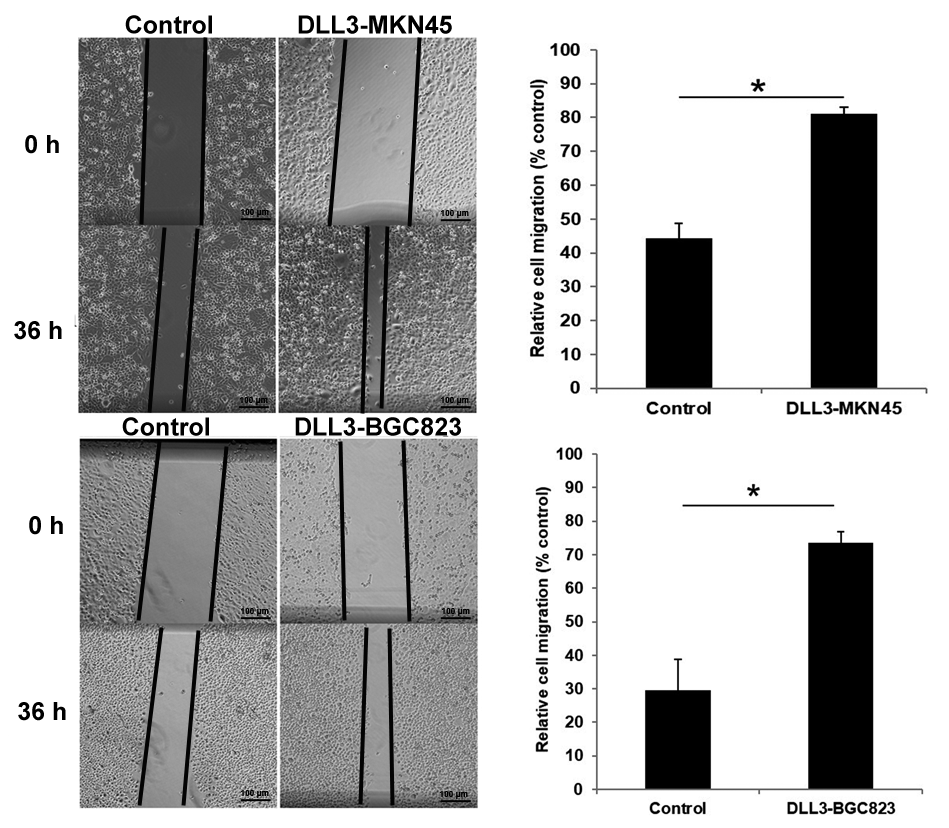

Supplement: goab052_Supplementary_Data [file goab052_supplementary_data.zip › GR-2021-002_Supplymentary Figure 1.tif]

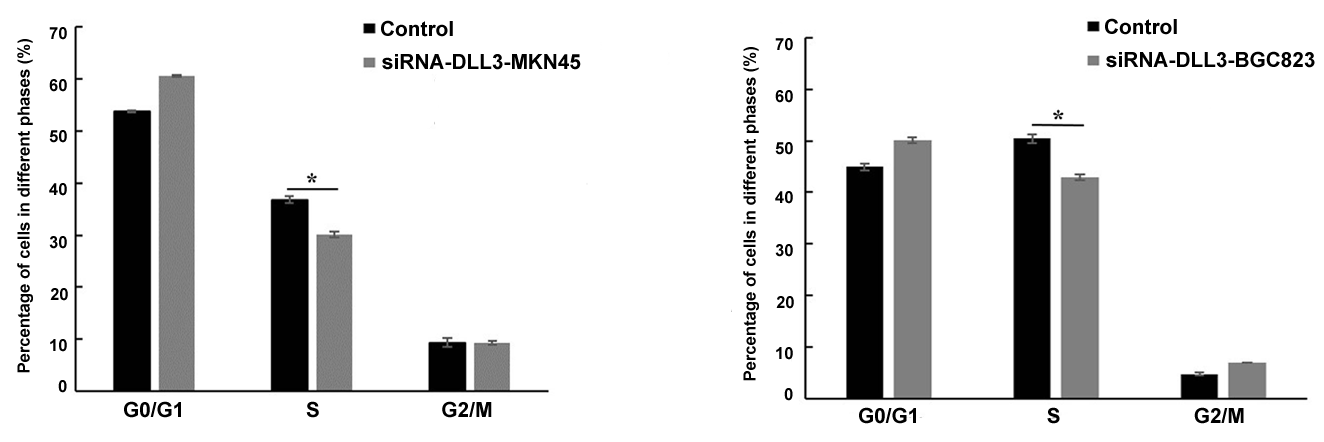

Supplement: goab052_Supplementary_Data [file goab052_supplementary_data.zip › GR-2021-002_Supplymentary Figure 2.tif]
